# Maintenance of genome stability in plants: repairing DNA double strand breaks and chromatin structure stability

**DOI:** 10.3389/fpls.2014.00487

**Published:** 2014-09-23

**Authors:** Sujit Roy

**Affiliations:** Protein Chemistry Laboratory, Department of Chemistry, Bose InstituteKolkata, India

**Keywords:** plant genome stability, environmental and genotoxic stress, DNA damage response, double strand breaks, chromatin remodeling

## Abstract

Plant cells are subject to high levels of DNA damage resulting from plant’s obligatory dependence on sunlight and the associated exposure to environmental stresses like solar UV radiation, high soil salinity, drought, chilling injury, and other air and soil pollutants including heavy metals and metabolic by-products from endogenous processes. The irreversible DNA damages, generated by the environmental and genotoxic stresses affect plant growth and development, reproduction, and crop productivity. Thus, for maintaining genome stability, plants have developed an extensive array of mechanisms for the detection and repair of DNA damages. This review will focus recent advances in our understanding of mechanisms regulating plant genome stability in the context of repairing of double stand breaks and chromatin structure maintenance.

## DNA DOUBLE STRAND BREAKS AND GENOME INSTABILITY

Plants, with their intrinsic immobility and obligatory exposure to sunlight for energy, are constantly facing the tremendous challenge of maintaining the genome integrity which is under continuous assault from environmental factors like solar UV and ionizing radiation, high soil salinity, drought and desiccation, chemical mutagens, and free radicals or alkylating agents generated by endogenous processes (Roy et al., 2009, 2013a; Tuteja et al., 2009; Waterworth et al., 2011; Yoshiyama et al., 2013). These agents cause variety of DNA damages including DNA base oxidation and alkylation, formation of pyrimidine dimers and abasic sites, single and double strand breaks (SSBs and DSBs), DNA inter-strand cross links and therefore seriously threat the integrity of plant genome. Lesions in the DNA, contributed by various damaging agents, may result in changes in both the chemical and physical structures of DNA and thus generate both cytotoxic and genotoxic effects, adversely affecting plant growth and development (Balestrazzi et al., 2011). Therefore, to survive under frequent and extreme environmental stress conditions, plant cells have evolved with highly efficient and wide-ranging mechanisms for the detection and repair of DNA damage to eliminate the chances of permanent genetic alterations and to maintain genome stability for faithful transfer of genetic information over generations (West et al., 2004; Kozak et al., 2009; Roy et al., 2011).

Among the various forms of DNA lesions, DSBs in DNA double helix are considered as one of the major form of DNA damage (West et al., 2004). In addition to genotoxic stress, which frequently induces DSBs, error prone DNA replication and defective repair of SSB or collapsed replication forks during trans lesion synthesis and steric stresses during DNA unwinding may also result in the formation of DSBs (Kuzminov, 2001). DSBs in the actively dividing plant tissues like shoot or root apical meristem (SAM and RAM) severely affect plant growth since DNA synthesis events or progression through cell division with unrepaired DSBs often results in chromosomal aberrations at the structural levels leading into loss of chromosome fragments (deletions), insertions, and chromosome fusions. Such aberrant chromosomal structures eventually severely affect plant growth and development due to inhibition of DNA replication and transcription which in turn results in loss of cell viability (Fulcher and Sablowski, 2009; Waterworth et al., 2011).

Efficient detection, activation of cell-cycle checkpoint function and rapid repair of DSBs in the genome is crucial for the survival of all organisms including plants (Puchta, 2005). The DSBs are repaired by two fundamental mechanisms: the homologous recombination (HR) and the non-homologous-end joining (NHEJ) pathway. The HR pathway is mediated by the proteins of RAD52 epistasis groups RAD51, RAD52, RAD54, RAD55, RAD57 and the MRN complex, comprising of MRE11, RAD50 and NBS1 (Symington, 2002). HR pathway requires an intact copy of the homologous DNA duplex for the formation of a heteroduplex for repairing the damaged strand using the non-damaged region as a template (Barzel and Kupiec, 2008). DSB repair via HR is commonly utilized in bacterial and yeast cells, depending on the availability of sequence homology. However, in eukaryotes, including mammals and plants, HR mediated DSB repair is crucial during the early stages of gamete formation in meiotic cells where a programmed induction of DSBs initiates homologous chromosome pairing and recombination (Edlinger and Schlogelhofer, 2011).

In mammals and plants with large and complex genomes, majority of DSBs in somatic cells are repaired via the NHEJ pathway (West et al., 2004; Puchta, 2005), in which the broken ends of double stranded DNA are directly joined irrespective of sequence homology. Thus, NHEJ repair is error-prone but represents the predominant DSB repair pathway during G1 to early S-phase of cell cycle. However this pathway has also been found to be functional throughout the cell cycle (Abe et al., 2009). In NHEJ repair, the KU70/80 complex binds to the DNA ends at the site of DSBs in the double stranded DNA. Broken ends are then processed by the MRN complex for making the ends suitable substrate for joining by the activity of DNA ligase IV and XRCC4. The gap filling synthesis requires involvement of DNA polymerase λ (Pol λ), the sole member of family X DNA polymerase in plants (Roy et al., 2013b).

## CELLULAR RESPONSE TO DNA DOUBLE STRAND BREAKS

Cellular responses to DSBs in the DNA are initiated by activation of a complex damage response pathway which includes the detection of DSBs, followed by signaling to regulate the mechanisms governing cell cycle progression, programmed cell death, and direct activation of DNA repair pathways (Zhou and Elledge, 2000). The molecular components of the HR and NHEJ mediated DSB repair pathways are highly conserved among eukaryotes, and previous studies have revealed requirements of both these pathways for DSB repair in plants (Bray and West, 2005). The major components of DSB detection in plants include the KU70–KU80 complex, which has high affinity for broken DNA ends and also acts as a core component of the NHEJ pathway (West et al., 2004). In addition, the multiprotein MRN complex has also been implicated in DSB detection and shown to be involved in both NHEJ- and HR-mediated DSB repair (Amiard et al., 2010).

**FIGURE 1:**
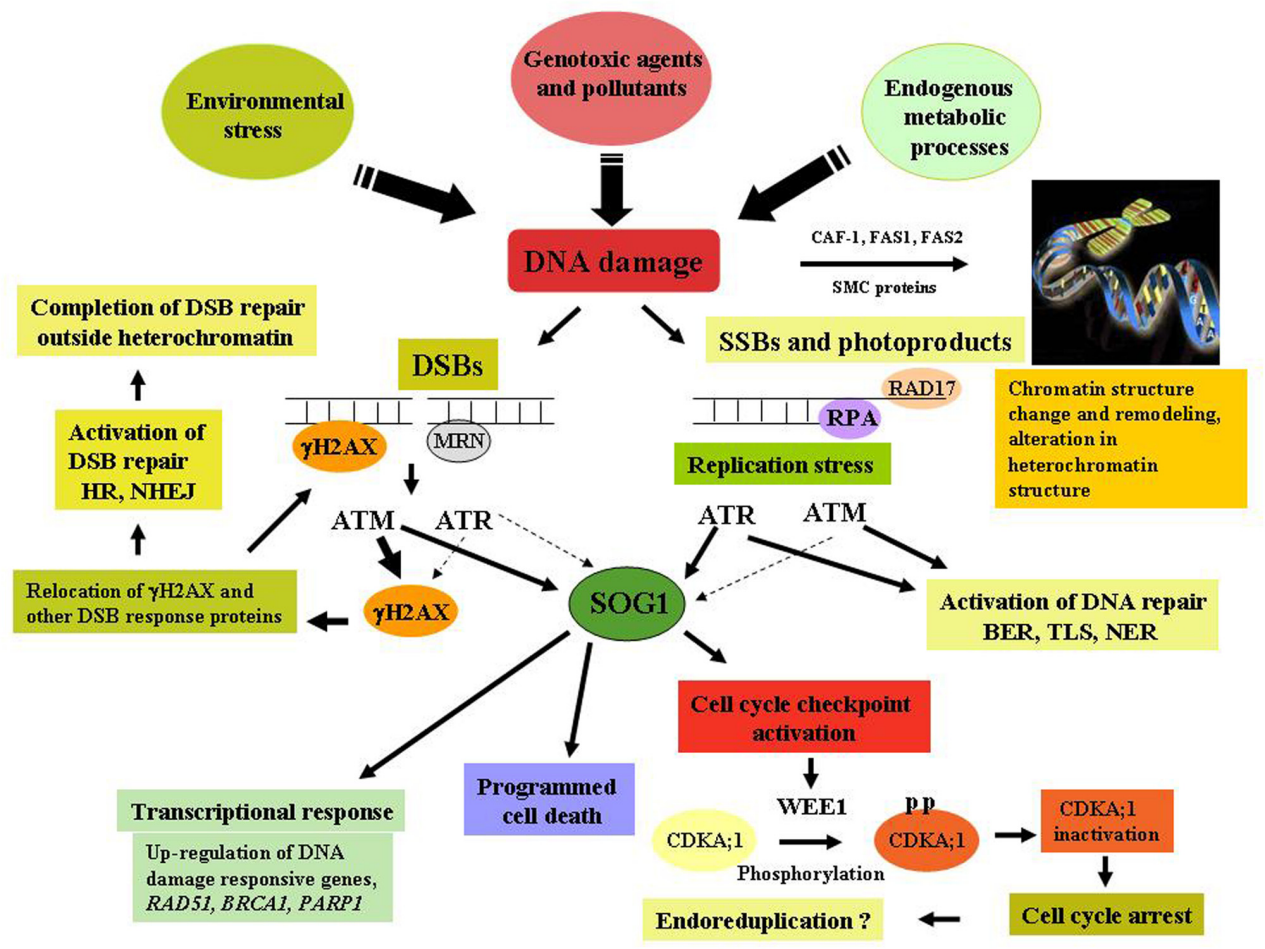
**DNA damage response and chromatin remodeling activity in plants.** Detection of DNA damage by the sensors- MRN complex, followed by the subsequent transduction of signals carried out by ataxia telangiectasia mutated (ATM) and ATR and Rad3-related (ATR) through phosphorylation of various target proteins including Histone H2AX, Chk1, Chk2, resulting in the activation of DNA repair, cell-cycle checkpoint function, programmed cell death via SUPPRESSOR OF GAMMA RESPONSE 1 (SOG1), a plant-specific transcription factor plays key role in DNA damage signaling. DNA damage also results in the change in chromatin structure, activation of remodeling activities and alteration of heterochromatin mediated by the activity of CAF-1, FAS1 and -2, and SMC proteins. Thick arrows indicate a major role, while thin arrows indicate small effects.

In eukaryotes, cellular response to DNA damage is governed by the two key regulators, ataxia telangiectasia mutated (ATM) and ATM and Rad3-related (ATR) kinases which are phosphoinositide-3-kinase-related protein kinases (PIKKs; Bradbury and Jackson, 2003), regulating cell cycle progression and activation of DNA repair pathways in response to DNA damage. ATM has been shown to be mainly activated by genotoxins which generate DSBs (Lee and Paull, 2004), resulting in the up-regulation of large number of genes encoding factors involved in DNA repair processes, DSB signaling and cell cycle regulation, while down regulating expression of G2 and M-phase specific genes, leading to cell cycle arrest in response to DNA damage (Culligan et al., 2006). Conversely, ATR is more strongly activated in response to replication stress, resulting in the activation of cell-cycle checkpoint function. Like other eukaryotes, the activation of ATM and ATR kinases are crucial in plants in regulating the DNA damage signaling directly or indirectly through phosphorylation of multiple target proteins, including the phosphorylation of histone 2A isoforms H2AX, NBS1 and the other checkpoint associated protein kinases, including Chk1 (check point kinase) and Chk2 (Matsuoka et al., 2007). In *Arabidopsis*, a unique plant-specific transcription factor SUPPRESSOR OF GAMMA RESPONSE 1 (SOG1) has been shown to act as the central regulator in DNA damage response pathway and suggested to perform analogous functions to mammalian p53 in plant genome, involved in majority of plant’s response to DNA damage, such as transcriptional response, activation of cell cycle checkpoint and programmed death of stem cells (Yoshiyama et al., 2014; Figure 1).

## UNDERSTANDING THE LINK BETWEEN CHROMATIN STRUCTURE STABILITY AND DNA DOUBLE STRAND BREAK REPAIR IN THE CONTEXT OF PLANT GENOME STABILITY MAINTENANCE

Like other eukaryotes, plant genome is organized into chromatin which is the functional template for variety of fundamental biological processes, like DNA replication, transcription, repair, and recombination. Chromatin structure is crucial for genome stability and is constituted by the association of histone complexes with DNA to form nucleosomes. This step is regulated by two major pathways (Polo and Almouzni, 2006), one of which is dependent on histone gene repressor (HIRA) whereas the other pathway requires chromatin assembly factor-1 (CAF-1), which is tightly linked with DNA replication (Ramirez-Parra and Gutierrez, 2007). The CAF-1 chaperone, a heterotrimeric complex, comprising of FASCIATA 1 (FAS1), FAS2, and MULTICOPY SUPPRESSOR OF IRA1 (MSI1) subunits in *Arabidopsis* (Hennig et al., 2003), targets acetylated histone H3/H4 onto nascent DNA strand, allowing *de novo* assembly of nucleosomes (Polo and Almouzni, 2006). In mammals including human, CAF-1 is essential for cell cycle progression, while in *Arabidopsis* CAF-1 mutants are fully viable but display defects in meristem organization, as found in *fas1* and *fas2* mutants (Ramirez-Parra and Gutierrez, 2007). The distorted meristem structure due to loss of CAF-1 function results in characteristic growth fasciation. Interestingly such phenotypes were also reported in DSB repair pathway mutants, like *mre11* and *brca2* and in wild-type *Arabidopsis* following high doses of irradiation (Abe et al., 2009).

Global transcriptomic analyses in *Arabidopsis* have revealed that despite pleiotropic developmental defects, <2% of genes are transcriptionally deregulated in *Arabidopsis* CAF-1 mutants and within this a fairly high proportion of the genes are associated with DNA damage repair (Schönrock et al., 2006), indicating functional link between CAF-1 and thus chromatin structure stability and DNA damage response (Ramirez-Parra and Gutierrez, 2007). In *fas1* mutant, up regulated expression of the DNA damage responsive genes, like *RAD51*, *PARP1,* and *BRCA1* and *CYCB1;*1 have been demonstrated as a result of selective epigenetic changes in histone H3 acetylation and methylation in the promoters of these genes, but not because of global changes in chromatin modeling. Similar responses were also detected when wild-type *Arabidopsis* were subjected to DNA damaging agents, indicating that defects in chromatin assembly during S-phase and DNA damage signaling probably share part of the similar pathway via changing the epigenetic status of the target genes (Ramirez-Parra and Gutierrez, 2007). In *fas1* and *fas2* mutants, defects in chromatin assembly has also been shown to cause hypersensitive response toward genotoxic agents along with the increased basal levels of DSBs and constitutive activation signal for DNA damage response pathway, resulting in significant increase in spontaneous intrachromosomal recombination (Takeda et al., 2004; Endo et al., 2006). The activation of DNA damage response and the associated decrease of cell number in *fas1* mutant were found to be dependent on ATM kinase, one of the master controllers in DDR pathway (Hisanaga et al., 2013). *Arabidopsis* mutants, deficient in DNA replication factors, including Replication Protein A1 (RPA1) and Topoisomerase VI, display phenotypes of chromatin assembly mutants, such as constitutive activation of DNA damage response and in some cases loss of transcriptional gene silencing due to destabilization of heterochromatin (Elmayan et al., 2005; Breuer et al., 2007), as observed in mutants in CAF-1 complex (Ramirez-Parra and Gutierrez, 2007). In *Arabidopsis, BRU1* gene encodes a CAF. *Bru1* mutant plants showed hypersensitivity to genotoxic stress with constitutive activation of DNA damage response and loss of transcriptional gene silencing (Takeda et al., 2004), suggesting interesting cross talk points between chromatin assembly, DNA damage repair, and epigenetic inheritance. In addition, recent studies have revealed involvement of chromatin remodeling proteins in repair of DNA damages. In *Arabidopsis*, the histone acetyltransferases HAM1 and HAM2 participate in repair of UV-B induced DNA damage, suggesting importance of chromatin remodeling and histone acetylation during repair of UV-B induced DNA damage (Campi et al., 2012). In *Arabidopsis*, the key histone H3/H4 chaperone ANTI-SILENCING FUNCTION1 (ASF1) is involved in UV-B induced DNA damage repair (Lario et al., 2013). Together, these observations indicate that DNA repair in plants is regulated both at the genetic and epigenetic levels.

## RAPID REPAIR OF DOUBLE STRAND BREAKS IN PLANTS: STRUCTURAL MAINTENANCE OF CHROMOSOME PROTEINS

Non-homologous-end joining has been considered as the preferred pathway involved in the repair of majority of DSBs in higher plants. Interestingly, *Arabidopsis* NHEJ knockout mutants *ku80* and *lig4* were found to repair DSBs very rapidly, with comparable rates to wild-type plants, indicating the involvement of “classic NHEJ” independent novel backup pathway which probably regulate rapid repair of the majority of DSBs in plant cells. Rapid repair of DSBs in plants has been shown to be mediated by the plant ortholog of structure maintenance of chromosome proteins, MIM (AtSMC6/AtRAD18) and kleisin (AtRAD21.1; Kozak et al., 2009). The members of the STRUCTURAL MAINTENANCE OF CHROMOSOMES (SMC) family and the associated non-SMC factors play crucial role in the regulation of higher order chromatin structure in eukaryotes (Schubert, 2009). The SMC proteins contain characteristic ATPase activity and, along with the non-SMC proteins like kleisin subunits, form multiprotein complexes – cohesion, condensin, and the SMC5/6 complex (Watanabe et al., 2009). Cohesin, together with the SMC5/6 complex, is involved in DSB repair in G2 cells. In *Arabidopsis*, a subunit mutant of the cohesin complex, RAD21.1, displayed enhanced sensitivity to genotoxins with low DSB repair rates (Kozak et al., 2009). Homologs of additional cohesin establishment factors, including E2F target gene 1 (ETG) and CTF18, have been identified in *Arabidopsis* (Takahashi et al., 2010). The *etg* and *ctf18* mutants showed partial loss in chromatid cohesion, along with the constitutive activation of DNA damage response. The effect was more severe in the double mutant line (Takahashi et al., 2010). The involvement of cohesion establishment factor CHROMOSOME TRANSMISSION FIDELITY 7 (AtCTF7/ECO1) in DNA repair and cell division was established in *Arabidopsis*. The *ctf7-1* and *ctf7-2* mutants showed growth defects, poor anther development and sterility, deficiency in DNA repair and cell division with increased expression of DNA repair genes, such as *BRCA1* and *PARP2* (Bolanos-Villegas et al., 2013), demonstrating key role of cohesins for sister chromatid cohesion and DNA damage response in maintaining plant genome stability.

## OUTLOOK

With the completion of *Arabidopsis* genome project, understanding the link between DSB repair and chromatin structure maintenance has become the subject of intense study over the past few years. The above discussion summarizes recent advancement in our understanding of the link connecting chromatin structure stability with DNA damage response for the genetic and epigenetic maintenance of genome stability in plants. Considering the impact of global change in climate on plant growth, development, and productivity, further research in this area in future will provide meaningful insight about how plants maintain genome stability under environmental and genotoxic stresses.

## Conflict of Interest Statement

The author declares that the research was conducted in the absence of any commercial or financial relationships that could be construed as a potential conflict of interest.
